# Magnetic resonance imaging of penile paraffinoma: case report

**DOI:** 10.1186/1471-2342-14-39

**Published:** 2014-12-08

**Authors:** Luigi Cormio, Giuseppe Di Fino, Carmen Scavone, Oscar Selvaggio, Paolo Massenio, Francesca Sanguedolce, Luca Macarini, Giuseppe Carrieri

**Affiliations:** Department of Urology and Renal Transplantation, University of Foggia, Via Luigi Pinto, 71122 Foggia, Italy; Department of Radiology, University of Foggia, Foggia, Italy; Department of Pathology, University of Foggia, Foggia, Italy

**Keywords:** Lipogranuloma, Penis, Mineral oils, Diagnosis, MRI

## Abstract

**Background:**

Penile paraffinoma is a well-known delayed complication of paraffin oil injection into the penis for penile girth augmentation but its MRI features have not been previously described.

**Case presentation:**

A 35-year-old Ukraine man presented with erectile dysfunction, voiding difficulty and an irregular, hard and painful penile mass that had progressively grown over the last year. He reported having received, seven years before, several penile injections of paraffin oil for penile girth augmentation. On physical examination, the mass was tender, poorly delimited, and involved the whole penile shaft and the cranial part of the scrotum. Preoperative MRI, performed to determine the extent of tissue to be removed and the possibilities of penile reconstruction, showed a newly-formed homogeneous tissue, compressing but not infiltrating Buck’s fascia, iso-hypointense relative to muscle on T1-weighted sequences, and with a low signal intensity at T2-weighted sequences. On T1-weighted fat suppressed sequences, it did not enhance with contrast administration. MRI data were confirmed by surgical findings, as the newly-formed scar tissue did not infiltrate Buck’s fascia. Pathology confirmed the diagnosis of penile paraffinoma.

**Conclusion:**

MRI seems to provide an adequate imaging of the histological events occurring after injection of paraffin oil in the subcutaneous tissues. Penile paraffinoma remains a clinical diagnosis, but MRI features may be helpful in planning an adequate surgical strategy and, in selected cases, establishing the differential diagnosis with other penile diseases, including cancer.

## Background

In the first half of the 20th century, subcutaneous injection of liquid paraffin has widely been used worldwide [1] for cosmetic purposes such as filling of wrinkles or cheeks, facial deformities, baldness [2], as well as to produce an artificial augmentation of muscle, breast [3] and penile shaft [4]. The potential destructive complications of such practice, however, have been reported since 1906 [5]. The most relevant acute complication is infection; delayed complications, conversely, are linked to a granulomatous foreign body reaction named paraffinoma [4, 6].

Penile paraffinoma may present with penile scarring and deformity, abscess formation, ulceration, erectile dysfunction, painful intercourse, and voiding problems [4] up to acute urinary retention [7]. Although all such problems are well known, penile injection of paraffin remains a common means of increasing penile girth for Eastern Europe and Eastern Asia people*.* Reasons for this unreasonable practice include, on one hand, the fact that it is easily available and easily performed by non-medical personnel or the patient himself, on the other hand, lack of standardized medical or surgical techniques for penile girth enhancement.

Most reported cases of penile paraffinoma focused on disease clinical presentation or on techniques for surgical excision and penile reconstruction [8, 9]. To our knowledge, the imaging features of penile paraffinoma have not been previously described. Therefore, we describe herein MRI findings in a case of penile paraffinoma and discuss their potential implications on differential diagnosis and surgical planning.

## Case presentation

A 35-year-old man emigrated from Ukraine presented to our Emergency Department with an irregular penile mass associated with erectile dysfunction and voiding difficulty. The patient reported that the penile mass had progressively grown over the last year up to become, in the last month, hard and painful. Physical examination revealed a tender, poorly delimited, subcutaneous indurated mass that extended on the whole surface of the penile shaft, from the coronal sulcus to the cranial part of the scrotum whereby the granulation tissue was palpable under the skin. The mass had no adherence to the overlying planes and was painful on palpation. There was no ulceration or inguinal lymphadenopathy.

**Figure 1:**
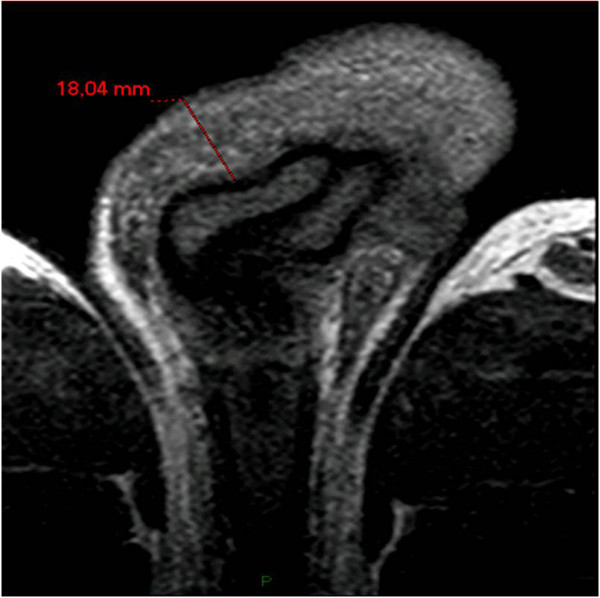
**Axial T1-weighted TSA MR imaging shows iso-hypointense 18 mm tissue (red line) surrounding the corpora cavernosa of the penis.**

**Figure 2:**
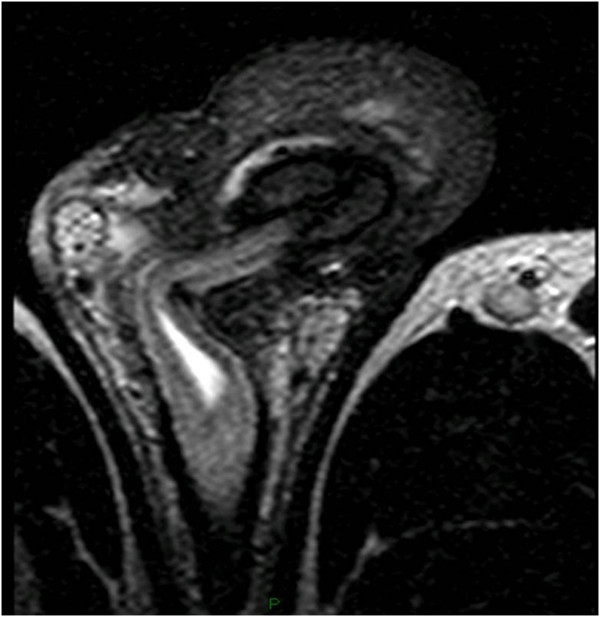
**Axial T2-weighted TSA MR imaging showing the fibrotic tissue that leads to a severe narrowing of the penile urethra with dilatation of the upstream tract.**

**Figure 3:**
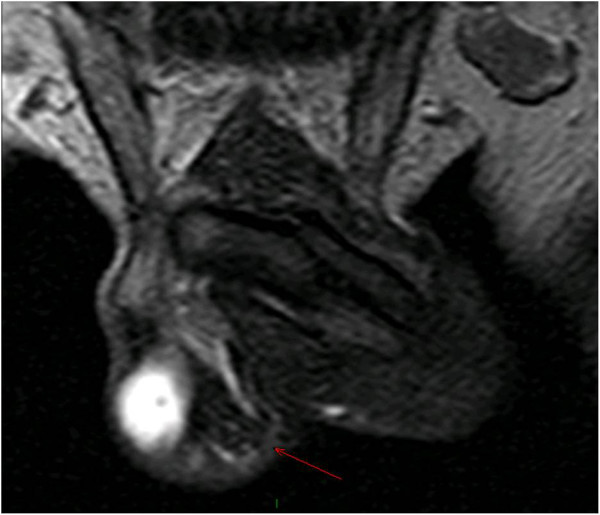
**Coronal T2-weighted TSA MR imaging showing fibrotic reaction (red arrow) extended in the connective tissue of the scrotal sac.**

There were no relevant findings in patient’s medical history and he reluctantly admitted that 7 years before he had received 6 injections of paraffin oil into the penis, performed by an untrained person, for penile enlargement purposes; therefore, penile paraffinoma was diagnosed. He was scheduled for definite surgical treatment involving complete removal of the newly-formed penile mass and penile shaft reconstruction. In view of the extent of tissue to be removed and the risk of scrotal tissue as well being involved by scarring, thus leading to its removal rather than its use for reconstruction, the patient underwent preoperative penile MRI to properly plan surgery.Penile MRI was performed with a 1.5 T whole body MR unit (Philips Achieva) with the patient supine. The penis was flexed dorsally against the lower abdomen and taped in position to reduce motion of the organ during the examination. A SENSE Torso coil (8 elements) was positioned on the pelvis. First, axial T2-weighted TSE (Turbo Spin Echo) images (TR/TE, 10111/120 ms; ETL, 20) and axial T1-weighted TSE images (889/12) were acquired. Then, coronal FFE T2-weighted images (500/13), sagittal and coronal TSE T2-weighted (6009/120) were acquired. Finally, axial T1-weighted GRE images (10,9/5,4) were acquired with fat suppression before and after a bolus injection of gadopentate dimegluine (0.5 mmol/mL; 0.2 mL/Kg). During all pulse sequences we used the following common parameters: a field of view of 260 mm, a slice thickness of 4 mm, and a matrix of 320 × 224. MRI showed a 18 mm thick lesion in the subcutaneous tissues of the penile shaft, surrounding the cavernous bodies up to spraining them in some points, causing a minimal distortion of the glans and a moderate narrowing of the bulbar urethra (Figure 1). Such lesion showed a homogeneous signal, iso-hypointense relative to muscle on T1-weighted sequences, and a low signal intensity at T2-weighted sequences (Figure 2). On T1-weighted fat suppressed sequences, it did not enhance with contrast administration. MRI features were therefore consistent with a fibrotic scar-tissue between dartos and Buck’s fascia, attached but not infiltrating the latter, which remained visible as a thin line hypointense in all pulse sequences. MRI also showed, in the cranial part of the scrotum, a granulation tissue with high signal intensity on T2-weighted turbo spin-echo sequence, low signal intensity on T1-weighted turbo spin-echo sequence, and significant homogeneous contrast enhancement (Figure 3).

**Figure 4:**
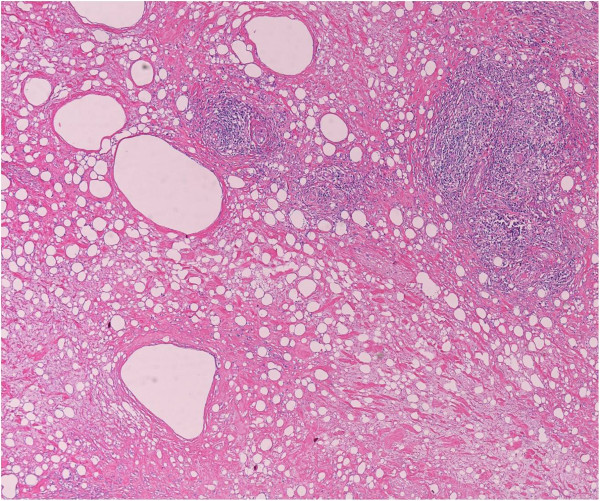
**Histological image of the removed tissue showing optical empty spaces indicative of the presence of large fat globules dissolved during the staining process, along with a heavy inflammation mainly consisting of lymphocytes and histiocytes, throughout the whole specimen.**

On the basis of these findings the patient was scheduled for surgical removal of the lesion, taking care to preserve Buck’s fascia and the underlying dorsal penile neurovascular bundle and to reconstruct with vital tissue the penile shaft. Postoperative course was uneventful; the urethral catheter was removed on the following morning and the patient discharged. At six weeks follow-up, he reported to be very satisfied with the cosmetic result as well as with his sexual function. Histological examination of the removed tissue confirmed the diagnosis of paraffinoma, showing optical empty spaces indicative of the presence of large fat globules dissolved during the staining process, along with a heavy inflammation mainly consisting of lymphocytes and histiocytes, throughout the whole specimen (Figure 4).

## Conclusion

There is clinical and histological evidence [10, 11] that injection of paraffin oil into penile subcutaneous tissue promotes a slow foreign-body granulomatous reaction leading to conversion of the liquid mineral oil into a solid, fibrous-like tissue. The “early” MRI appearance of paraffin oil into penile shaft has been described by Picozzi et al. [12] who reported that, shortly (48 hours) after injection, the oil displays hyperintensity on both T1- and T2-weighted sequences. Herein we provide the first description of MRI findings in case penile paraffinoma, a “late” event following paraffin oil injection into the penis. Using the described MRI sequences, we found that, like fibrous tissue, penile paraffinoma displayed low to intermediate intensity on both T1- and T2- weighted sequences, with no contrast enhancement. Taking these findings together, it can be stated that MRI provides an adequate imaging of the histological events occurring after injection of paraffin in the penile subcutaneous tissue.

An interesting finding of our report is that, in case of paraffinoma, the newly-formed fibrotic tissue does not jeopardizes the ability of MR imaging to evaluate the normal penile structures, particularly Buck’s fascia and neurovascular bundle. As a matter of fact, surgical exploration confirmed our MRI findings of paraffinoma fibrotic tissue attached but not infiltrating Buck’s fascia, which was visible as a thin line hypointense in all pulse sequences. This information is of great clinical relevance in planning the surgical strategy and in counseling patient’s about cosmetic and functional results of surgery.

Another interesting point is that, although penile paraffinoma remains a clinical diagnosis based on patient’s admittance of such practice, diseases such as thrombosis of dorsal penile vein, syphilis, herpes genitalis, Behcet and, last but not least, tumors [13] may occur independently on injection of paraffin into the penis. Therefore, further validation of the reported MRI findings of penile paraffinoma could provide valuable clues in the differential diagnosis with all such diseases.

Finally, our MRI findings of penile paraffinoma were consistent with MRI findings of paraffinomas in the cervico-facial area [14] as well as of breast paraffinoma [1, 15] somehow confirming the efficacy of MRI in providing an adequate imaging of the human body reaction to the injection of such oil.

In conclusion, though penile paraffinoma remains a clinical diagnosis, the MRI features described herein may be helpful in planning an adequate surgical strategy and, in selected cases, establishing the differential diagnosis with other penile diseases, including cancer.

## Consent

Written informed consent was obtained from the patient for publication of this report and any accompanying images. A copy of the written consents is available for review by the Editor-in-Chief of this journal.

## Abbreviations

MRI: Magnetic Resonance Imaging
TSE: Turbo Spin-Echo
T1W: T1-weighted
T2W: T2-weighted
TR: Repetition time
TE: Echo time
ETL: Echo train length.
